# Update: Characteristics of Patients in a National Outbreak of E-cigarette, or Vaping, Product Use–Associated Lung Injuries — United States, October 2019

**DOI:** 10.15585/mmwr.mm6843e1

**Published:** 2019-11-01

**Authors:** Erin D. Moritz, Lauren B. Zapata, Akaki Lekiachvili, Emily Glidden, Francis B. Annor, Angela K. Werner, Emily N. Ussery, Michelle M. Hughes, Anne Kimball, Carla L. DeSisto, Brandon Kenemer, Mays Shamout, Macarena C. Garcia, Sarah Reagan-Steiner, Emily E. Petersen, Emily H. Koumans, Matthew D. Ritchey, Brian A. King, Christopher M. Jones, Peter A. Briss, Lisa Delaney, Anita Patel, Kara D. Polen, Katie Sives, Dana Meaney-Delman, Kevin Chatham-Stephens, Lung Injury Response Epidemiology/Surveillance Group, Lung Injury Response Epidemiology/Surveillance Task Force, Adebola Adebayo, Jennifer Adjemian, Minal Amin, Jose Aponte, Grace Appiah, Christina Armatas, Melissa Arons, Sukhshant Kaur Atti, Michelle Banks, Vaughn Barry, Amy Board, Tegan Boehmer, John Bowyer, Lauren Boyle-Estheimer, Diane Browning, David Bui, Jordan Cates, Gyan Chandra, Karen Chang, Jennifer Chevinsky, Katelyn Chiang, Pyone Cho, George Cone, Matthew Cone, Kristen Cowan, Augustina Delaney, Lindsey Duca, Angela Dunn, Shideh Delrahim Ebrahim-Zadeh, Jeff Engel, Molly Evans, Victoria Fields, Aaron Fleishauer, Jennifer Freed, Allison Gately, Isaac Ghinai, Caitlin Green, Janet Hamilton, Arianna Hanchey, Kathleen Hartnett, Amy Heinzerling, Dessica Hodges, Brooke Hoots, Asad Islam, Mia Israel, Yunho Jang, Sumera Jiva, Jona Johnson, Charlotte Kaboré, Emily Kiernan, Uzay Kirbiyik, Hannah Kisselburgh, Mimisha Kothari, Vikram Krishnasamy, Mohammed Lamtahri, Michael Landen, Jennifer Layden, Mark Layer, Ruth Lynfield, Kristen Marshall, Keegan McCaffrey, Eva McLanahan, Jonathan Meiman, Christina Mikosz, Maureen Miller, Roger Mir, Yousra Mohamoud, Livia Navon, Varsha Neelam, David Nitschke, Rashid Njai, Kevin O’Laughlin, Samantha Olson, Cria Perrine, Cassandra Pickens, Mary Pomeroy, Aaron Kite Powell, Ian Pray, Tia Rogers, Nicole Roth, Phillip Salvatore, Caroline Schrodt, Sarah Shafer, Dhara Shah, Raschelle Smalley, Steven Sumner, Lauren Tanz, Mark Tenforde, Vilma Thomas, Megan Toe, Kate Varela, Alana Vivolo, Jason Wilken, Peter Yang, Anna Yousaf

**Affiliations:** ^1^National Center for Environmental Health, CDC; ^2^National Center for Chronic Disease Prevention and Health Promotion, CDC; ^3^National Center for Injury Prevention and Control, CDC; ^4^National Center on Birth Defects and Developmental Disabilities, CDC; ^5^Epidemic Intelligence Service, CDC; ^6^National Center for HIV/AIDS, Viral Hepatitis, STD, and TB Prevention, CDC; ^7^Center for Surveillance, Epidemiology, and Laboratory Services, CDC; ^8^National Center for Emerging and Zoonotic Infectious Diseases, CDC; ^9^National Institute for Occupational Safety and Health, CDC; ^10^National Center for Immunization and Respiratory Disease, CDC.; National Center for Immunization and Respiratory Diseases, CDC; Center for Surveillance, Epidemiology, and Laboratory Services, CDC; National Center for Immunization and Respiratory Diseases, CDC; Center for Surveillance, Epidemiology, and Laboratory Services, CDC; National Center for Emerging and Zoonotic Infectious Diseases, CDC; California Department of Public Health; Epidemic Intelligence Service, Center for Global Health, CDC; Agency for Toxic Substances and Disease Registry, CDC; National Center for Immunization and Respiratory Diseases, CDC; Epidemic Intelligence Service, National Center for Injury Prevention and Control, CDC; Epidemic Intelligence Service, National Center for Injury Prevention and Control, CDC; National Center for Environmental Health, CDC; National Center for Emerging and Zoonotic Infectious Diseases, CDC; National Center for Chronic Disease Prevention and Health Promotion, CDC; Northrop Grumman; Epidemic Intelligence Service, National Center for Environmental, CDC; Epidemic Intelligence Service, National Center for Immunization and Respiratory Diseases, CDC; National Center for Chronic Disease Prevention and Health Promotion, CDC; Epidemic Intelligence Service, National Center for Chronic Disease Prevention and Health Promotion, CDC; Epidemic Intelligence Service, National Center for Chronic Disease Prevention and Health Promotion, CDC; National Center for Chronic Disease Prevention and Health Promotion, CDC; National Center for Chronic Disease Prevention and Health Promotion, CDC; National Center for Immunization and Respiratory Diseases, CDC; Council of State and Territorial Epidemiologists; , National Center for Environmental Health, CDC; G2S Corporation; Epidemic Intelligence Service, National Center for Chronic Disease Prevention and Health Promotion, CDC; Utah Department of Health; National Center for Environmental Health, CDC; Council of State and Territorial Epidemiologists; National Center for Injury Prevention and Control, CDC; Epidemic Intelligence Service, National Center on Birth Defects and Developmental Disabilities CDC; North Carolina Department of Health and Human Services; Agency for Toxic Substances and Disease Registry, CDC; National Center for Injury Prevention and Control, CDC; Illinois Department of Health; National Center on Birth Defects and Developmental Disabilities, CDC; Council of State and Territorial Epidemiologists; National Center for Environmental Health, CDC; Center for Surveillance, Epidemiology, and Laboratory Services, CDC; California Department of Public Health; Mercer University; National Center for Injury Prevention and Control, CDC; Center for Surveillance, Epidemiology, and Laboratory Services, CDC; Council of State and Territorial Epidemiologists; National Center for Immunization and Respiratory Diseases, CDC; Mercer University; Agency for Toxic Substances and Disease Registry, CDC; National Center for Chronic Disease Prevention and Health Promotion, CDC; Office of the Director, CDC; Center for Surveillance, Epidemiology, and Laboratory Services, CDC; National Center for Emerging and Zoonotic Infectious Diseases, CDC; Center for Surveillance, Epidemiology, and Laboratory Services, CDC; National Center for Injury Prevention and Control, CDC; Center for Surveillance, Epidemiology, and Laboratory Services, CDC; New Mexico Department of Health; Illinois Department of Public Health; National Center for Environmental Health, CDC; Minnesota Department of Health; Epidemic Intelligence Service, Center for Surveillance, Epidemiology, and Laboratory Services, CDC; Utah Department of Health; Office of the Director, CDC; Wisconsin Department of Health; National Center for Injury Prevention and Control, CDC; Epidemic Intelligence Service, National Center for Chronic Disease Prevention and Health Promotion, CDC; Center for Surveillance, Epidemiology, and Laboratory Services, CDC; National Center for Chronic Disease Prevention and Health Promotion, CDC; Illinois Department of Public Health; National Center on Birth Defects and Developmental Disabilities, CDC; , Center for Surveillance, Epidemiology, and Laboratory Services, CDC; Office of the Deputy Director for Non-Infectious Diseases, Office of the Director, CDC; Epidemic Intelligence Service, National Center for Emerging and Zoonotic Infectious Diseases, CDC; G2S Corporation; National Center for Chronic Disease Prevention and Health Promotion, CDC; National Center for Injury Prevention and Control, CDC; Epidemic Intelligence Service, National Center for Emerging and Zoonotic Infectious Diseases, CDC; Center for Surveillance, Epidemiology, and Laboratory Services, CDC; Wisconsin Department of Health Services; Epidemic Intelligence Service, National Center for Injury Prevention and Control, CDC; Eagle Medical Services; Epidemic Intelligence Service, National Center for Injury Prevention and Control, CDC; Epidemic Intelligence Service, National Center for Emerging and Zoonotic Infectious Diseases, CDC; National Center for Immunization and Respiratory Diseases, CDC; Council of State and Territorial Epidemiologists; Mercer University; National Center for Injury Prevention and Control, CDC; North Carolina Department of Health and Human Services; Center for Surveillance, Epidemiology, and Laboratory Services, CDC; National Center for Chronic Disease Prevention and Health Promotion, CDC; Council of State and Territorial Epidemiologists; Epidemic Intelligence Service, National Institute for Occupational Safety and Health; National Center for Injury Prevention and Control, CDC; California Department of Public Health; National Center for Chronic Disease Prevention and Health Promotion, CDC; National Center for Immunization and Respiratory Diseases, CDC

*On October 28, 2019, this report was posted as an *MMWR* Early Release on the *MMWR* website (https://www.cdc.gov/mmwr).*

CDC, the Food and Drug Administration, state and local health departments, and other public health and clinical stakeholders are investigating a national outbreak of electronic-cigarette (e-cigarette), or vaping, product use–associated lung injury (EVALI) (*1*). As of October 22, 2019, 49 states, the District of Columbia (DC), and the U.S. Virgin Islands have reported 1,604 cases of EVALI to CDC, including 34 (2.1%) EVALI-associated deaths in 24 states. Based on data collected as of October 15, 2019, this report updates data on patient characteristics and substances used in e-cigarette, or vaping, products (*2*) and describes characteristics of EVALI-associated deaths. The median age of EVALI patients who survived was 23 years, and the median age of EVALI patients who died was 45 years. Among 867 (54%) EVALI patients with available data on use of specific e-cigarette, or vaping, products in the 3 months preceding symptom onset, 86% reported any use of tetrahydrocannabinol (THC)-containing products, 64% reported any use of nicotine-containing products, and 52% reported use of both. Exclusive use of THC-containing products was reported by 34% of patients and exclusive use of nicotine-containing products by 11%, and for 2% of patients, no use of either THC- or nicotine-containing products was reported. Among 19 EVALI patients who died and for whom substance use data were available, 84% reported any use of THC-containing products, including 63% who reported exclusive use of THC-containing products; 37% reported any use of nicotine-containing products, including 16% who reported exclusive use of nicotine-containing products. To date, no single compound or ingredient used in e-cigarette, or vaping, products has emerged as the cause of EVALI, and there might be more than one cause. Because most patients reported using THC-containing products before symptom onset, CDC recommends that persons should not use e-cigarette, or vaping, products that contain THC. In addition, because the specific compound or ingredient causing lung injury is not yet known, and while the investigation continues, persons should consider refraining from the use of all e-cigarette, or vaping, products.

State health departments, the Council of State and Territorial Epidemiologists Vaping Associated Pulmonary Injury Epidemiology Task Force, and CDC developed and disseminated surveillance case definitions* and data collection tools (i.e., patient interview and medical record abstraction forms) to monitor and track cases beginning in August 2019. Some states are using these tools, whereas others elected to use state-specific tools. States and jurisdictions routinely report the number of confirmed and probable EVALI cases to CDC on a voluntary basis and, when available, include data from medical record abstractions and patient interviews. Proxies (e.g., spouses or parents) were interviewed if patients were too ill or if they had died. Most states and jurisdictions report the number of cases to CDC as case status is determined; however, it can take up to several weeks to complete and submit information from interview and medical record abstraction. This report provides updated data on patient demographic characteristics; substances used in e-cigarette, or vaping, products; and characteristics of EVALI patients who died, based on cases reported to CDC with available interview and medical record abstraction data as of October 15, 2019. The median ages of patients were compared across groups using the Wilcoxon rank-sum test. SAS statistical software (version 9.4; SAS Institute) was used for the analysis.

As of October 22, 2019, 49 states, DC, and the U.S. Virgin Islands had reported 1,604 cases of EVALI to CDC, including 34 (2.1%) EVALI-associated deaths in 24 states. Among 1,378 patients with confirmed or probable EVALI reported to CDC by October 15, 2019, with available data, 964 (70%) were male (Table). No cases in pregnant women were reported. Among 1,364 patients with information on age, the median age was 24 years (range = 13–75 years) and was similar among males (23 years) and females (25 years); 737 (54%) patients were aged <25 years, and 1,081 (79%) were aged <35 years. Among 383 EVALI patients with available information on race/ethnicity, 298 (78%) were non-Hispanic white, and 62 (16%) were Hispanic. Among 867 patients with available data on substances used, 749 (86%) reported any use of THC-containing products, and 552 (64%) reported any use of nicotine-containing products in the 3 months preceding symptom onset; 455 patients (52%) reported use of both THC-containing products and nicotine-containing products, 294 (34%) reported exclusive use of THC-containing products, and 97 (11%) reported exclusive use of nicotine-containing products. Twenty-one (2%) patients reported no use of THC- or nicotine-containing products.

**TABLE:** Characteristics of patients with electronic cigarette (e-cigarette), or vaping, product use–associated lung injury (EVALI) reported to CDC — United States, August–October 2019*

Characteristic	No. /Total No. (%^†^)		
	EVALI patients who survived	EVALI–associated deaths	All EVALI patients
**Sex**			
Male	947/1,349 (70)	17/29 (59)	964/1,378 (70)
Female	402/1,349 (30)	12/29 (41)	414/1,378 (30)
**Age group (yrs)**			
13–17	735/1,335 (55)^§^	2/29 (7)^§^	196/1,364 (14)
18–24			541/1,364 (40)
25–34	339/1,335 (25)	5/29 (17)	344/1,364 (25)
35–44	165/1,335 (12)	7/29 (24)	172/1,364 (13)
45–64	79/1,335 (6)	8/29 (28)	87/1,364 (6)
65–75	17/1,335 (1)	7/29 (24)	24/1,364 (2)
**Median age, yrs (range)**			
Overall	23 (13–72)	45 (17–75)	24 (13–75)
Male	23 (13–68)	55 (17–71)	23 (13–71)
Female	25 (13–72)	43 (27–75)	25 (13–75)
**Race/Ethnicity^¶^**			
White	283/365 (78)	15/18 (83)	298/383 (78)
Black or African American	22/365 (6)**	1/18 (6)**	9/383 (2)
American Indian or Alaska Native			4/383 (1)
Asian, Native Hawaiian, or other Pacific Islander			5/383 (1)
Other			5/383 (1)
Hispanic	60/365 (16)	2/18 (11)	62/383 (16)
**Substances used in e-cigarette, or vaping, products^ ††,§§^**			
THC-containing products, any use	733/848 (86)	16/19 (84)	749/867 (86)
Nicotine-containing products, any use	545/848 (64)	7/19 (37)	552/867 (64)
Both THC- and nicotine-containing products, any use	451/848 (53)	4/19 (21)	455/867 (52)
THC-containing products, exclusive use	282/848 (33)	12/19 (63)	294/867 (34)
Nicotine-containing products, exclusive use	94/848 (11)	3/19 (16)	97/867 (11)
No THC- or nicotine-containing products reported	21/848 (2)	0/19 (0)	21/867 (2)

**Abbreviation:** THC = tetrahydrocannabinol.

* Reported as of October 15, 2019.

^†^ Percentages might not add up to 100% because of rounding.

^§^ Data for the 13–17 and 18–24 age groups were combined to protect patient identity.

^¶^ Whites; blacks or African Americans; American Indians or Alaska Natives; Asians, Native Hawaiians and other Pacific Islanders; and Others were non-Hispanic. Hispanic persons could be of any race.

** Data for persons in the following race/ethnicity groups were combined to protect patient identity: black or African American; American Indian or Alaska Native, Asian, Native Hawaiian, or other Pacific Islander, and Other.

^††^ In the 3 months preceding symptom onset; categories not mutually exclusive.

^§§^ Data on both THC- and nicotine-containing product use required to be included.

Among the 29 EVALI-associated deaths reported to CDC as of October 15, 2019, 59% (17) were male; the median age was 45 years (range = 17–75 years) overall (Table), 55 years (range = 17–71 years) among males, and 43 years (range = 27–75 years) among females; the age difference between males and females was not statistically significant (p = 0.5). Patients who died were older than patients who survived (p<0.01). Among 19 EVALI patients who died and for whom data on substance use was available, the use of any THC-containing products was reported by patients or proxies for 84% (16), including 63% (12) who exclusively used THC-containing products. Use of any nicotine-containing products was reported for 37% (seven), including 16% (three) who exclusively used nicotine-containing products. Use of both THC- and nicotine-containing products was reported in four decedents.

## Discussion

Cases of EVALI continue to be reported to CDC as part of this national outbreak. Similar to previous reports at the national and state levels (*1*–*4*), most patients reported use of THC-containing products in the 3 months before symptom onset. Patients were predominantly aged <35 years, non-Hispanic white, and male. Patients with EVALI who died were older than patients who survived. Illnesses and deaths occurred across an age spectrum, from adolescents to older adults. Approximately half of cases, and two deaths, occurred in patients aged <25 years. Older adults were disproportionately represented among patients who died; only 2% of cases, but nearly 25% of deaths, occurred in patients aged >65 years. Further, any use of THC-containing products was reported for 86% of patients who survived and 84% of patients who died; exclusive use of THC-containing products was reported for 63% of EVALI patients who died and for 33% who survived.

Findings from this report, which is the largest analysis of EVALI patients to date, suggest that this outbreak continues to primarily affect young persons, highlighting the need to communicate the dangers of e-cigarette, or vaping, use particularly among youths and young adults. Although 2% of all EVALI patients were aged 65–75 years, 24% of deaths were in this age group; relevant tailored and targeted messaging might also be needed for this age group. Consistent with previously published reports (*1*–*4*), the data presented here suggest that THC-containing products are playing an important role in this outbreak. Further, reports from Illinois, Utah, and Wisconsin suggest that patients have typically obtained their THC-containing e-cigarette, or vaping, products through informal sources, such as friends or illicit in-person and online dealers, although local and regional differences in illicit THC supply and production might exist (*3*,*4*).

The findings in this report are subject to at least three limitations. First, data on substances used in e-cigarette, or vaping, products were self-reported or reported by proxies and might be subject to recall bias, as well as social desirability bias because nonmedical marijuana is illegal in many states. Therefore, underreporting might have occurred, particularly for patients who died and others whose information was provided by a proxy. Second, data on some variables, such as race/ethnicity, were missing for many patients, and conclusions based on these data might not be generalizable to the entire patient population. Finally, these data might be subject to misclassification of substance use for multiple reasons. Patients likely did not know the content of the e-cigarette, or vaping, products they used, and methods used to collect substance use data varied across states.

To date, no single compound or ingredient has emerged as the cause of EVALI, and there might be more than one cause. Because most patients report using THC-containing products before the onset of symptoms, CDC recommends that persons should not use e-cigarette, or vaping, products that contain THC. Persons should not buy any type of e-cigarette, or vaping, products, particularly those containing THC, off the street and should not modify or add any substances to e-cigarette, or vaping, products that are not intended by the manufacturer, including products purchased through retail establishments. In addition, because the specific compound or ingredient causing lung injury is not yet known, and while the investigation continues, persons should consider refraining from use of all e-cigarette, or vaping, products. E-cigarette, or vaping, products should never be used by youths, young adults, or women who are pregnant. Moreover, persons who do not currently use tobacco products should not start using e-cigarette, or vaping, products (*2*,*5*).

**Summary**

**What is already known about this topic?**

CDC and partners are investigating the ongoing outbreak of e-cigarette, or vaping, product use–associated lung injury (EVALI) in the United States, the District of Columbia, and one U.S. territory.

**What is added by this report?**

As of October 22, 2019, a total of 1,604 cases of EVALI, including 34 deaths, were reported to CDC. Based on data collected as of October 15, 2019, use of tetrahydrocannabinol (THC)-containing products in the 3 months preceding symptom onset was reported by 86% of patients. The median age of EVALI patients who survived was 23 years, and the median age of EVALI patients who died was 45 years.

**What are the implications for public health practice?**

Most EVALI patients report using THC-containing products before symptom onset. CDC recommends that persons should not use e-cigarette, or vaping, products containing THC. Because the specific compound or ingredient causing EVALI is not known, persons should consider refraining from use of all e-cigarette, or vaping, products.

## Footnotes

* https://www.cdc.gov/tobacco/basic_information/e-cigarettes/assets/2019-Lung-Injury-Surveillance-Case-Definition-508.pdf.
