# *Erratum:* Vol. 68, No. 43

**DOI:** 10.15585/mmwr.mm6850a6

**Published:** 2019-12-20

In the report “Update: Characteristics of Patients in a National Outbreak of E-cigarette, or Vaping, Product Use–Associated Lung Injuries — United States, October 2019,” on page 989, the list of contributors on the Lung Injury Response Epidemiology/Surveillance Task Force should have included **Samantha J. Lange, National Center for Chronic Disease Prevention and Health Promotion, CDC.**

